# Long-Term Outcomes of Early Autism Spectrum Disorder Screening: Prevalence and Diagnostic Stability in a Decade-Long Cohort from Türkiye

**DOI:** 10.3390/brainsci16010062

**Published:** 2025-12-31

**Authors:** İbrahim Selçuk Esin, Esen Yıldırım Demirdöğen, Mehmet Akif Akıncı, Bahadır Turan, Gülsüm Tuğba Korkmaz Ürük, İlknur İbili Ucuz, Onur Burak Dursun

**Affiliations:** 1 Department of Child and Adolescent Psychiatry, Faculty of Medicine, Trabzon University, Trabzon 61000, Türkiye; onurburak007@yahoo.com; 2 Department of Child and Adolescent Psychiatry, Faculty of Medicine, Atatürk University, Erzurum 25100, Türkiye; esenyildirim08@hotmail.com (E.Y.D.); akinci.mehmetakif@gmail.com (M.A.A.); gtugbakorkmaz@gmail.com (G.T.K.Ü.); 3 Department of Child and Adolescent Psychiatry, Faculty of Medicine, Karadeniz Teknik University, Trabzon 61000, Türkiye; bhdrturan@gmail.com; 4 Department of Child and Adolescent Psychiatry, Faculty of Medicine, İnonü University, Malatya 44100, Türkiye; ilknur_27@yahoo.com

**Keywords:** autism spectrum disorder, prevalence, diagnostic stability, longitudinal study

## Abstract

Objective: This study aimed to provide a reliable estimate of early childhood autism spectrum disorder (ASD) prevalence in Türkiye and to examine diagnostic stability and developmental trajectories through a ten-year longitudinal follow-up incorporating systematic early screening, structured parent-child observations, and repeated diagnostic assessments. Methods: A total of 1981 children aged 18-48 months were screened using the M-CHAT-R/F. Children who screened positive underwent an initial clinical assessment, including a family interview and structured parent-child observation. Those identified as at risk were referred for DSM-5-TR-based diagnostic evaluation by expert clinicians. Children diagnosed with ASD or classified as at risk were enrolled in a structured ten-year follow-up program. Results: Of the 1981 screened children, 27 (1.4%) were identified as at risk. Nine children (33.3% of at-risk; 0.45% of the total sample) received an ASD diagnosis following comprehensive evaluation. All retained their diagnosis during the 18-month follow-up. By the tenth year, two additional children from the at-risk group were diagnosed, bringing the total number of ASD cases to 11. Conclusions: These findings demonstrate that structured, multi-stage screening and diagnostic procedures are feasible and effective for early ASD identification in Türkiye. High diagnostic stability supports the reliability of early clinician-led assessments, while later-emerging cases highlight the importance of long-term monitoring of at-risk children.

## 1. Introduction

Autism spectrum disorder (ASD) is a neurodevelopmental condition characterized by persistent deficits in social communication and interaction, atypical sensory processing, and restricted, repetitive patterns of behavior, interests, or activities. It is widely regarded as a heterogeneous condition, demonstrating substantial variability in symptom presentation, severity, and required levels of support across individuals [1,2]. Accurate epidemiological estimates are essential for effective service planning and the appropriate allocation of resources for individuals with ASD, and are typically informed by both screening data and diagnostic assessments [3,4].

Systematic early screening is essential for the timely identification and intervention of children at risk for ASD. Autism-specific screening tools help detect children who require further evaluation, and the American Academy of Pediatrics recommends routine standardized screening at 18 and 24 months [5,6,7]. Evidence consistently shows that early screening accelerates the diagnostic process; for instance, one study reported that screened toddlers received an ASD diagnosis approximately 20 months earlier than their non-screened peers [8,9,10]. Several countries have currently implemented nationwide screening programs to promote the early detection of ASD. In Türkiye, one of the countries implementing such programs, a study including over 1.8 million children demonstrated that systematic screening effectively identifies children at risk and plays a critical role in early intervention strategies [11,12,13,14,15].

The prevalence of ASD has shown a consistent upward trend, particularly over the past two decades [16,17]. Since 2000, the Autism and Developmental Disabilities Monitoring Network has released biennial estimates of ASD prevalence among 8-year-old children in the United States. During this period, reported prevalence has risen markedly from 1 in 150 children in 2000 to 1 in 36 in 2020. The most recent data from the Centers for Disease Control and Prevention estimate the prevalence to be approximately 1 in 31 children [18,19,20]. Despite these increases, substantial variability in study methodologies such as differences in case definitions, sampling frameworks, and diagnostic criteria continues to hinder cross-study comparisons and complicates the development of a unified understanding of ASD prevalence globally. Moreover, ongoing debate remains as to whether the observed rise reflects a true increase in incidence or is primarily driven by methodological factors, including heightened public and professional awareness, diagnostic substitution, and evolving diagnostic practices [5,21]. Limited longitudinal follow-up of at-risk individuals has been identified as a key factor contributing to ongoing debates regarding autism prevalence [22,23,24].

Numerous studies and systematic reviews have examined ASD prevalence across Asia and Europe [25,26,27]; however, most rely on cross-sectional data, with limited longitudinal follow-up to track developmental trajectories over time [28]. Türkiye, a transcontinental country with one of the youngest populations globally, currently lacks nationally representative prevalence estimates [29]. This study addresses both the international and national gaps by investigating ASD prevalence in Turkish children through a ten-year longitudinal follow-up, aiming to identify children at risk using the Modified Checklist for Autism in Toddlers, Revised with Follow-Up (M-CHAT-R/F) and structured parent-child interaction observations, monitor the developmental trajectories of at-risk and diagnosed children, and establish a reliable estimate of ASD prevalence through repeated diagnostic assessments and comprehensive follow-up.

## 2. Materials and Methods

### 2.1. Study Design and Ethics Approval

This prospective cohort study was approved by the Clinical Research Ethics Committee and conducted within the Early Psychosocial Problems Identification Model (EPSIM) framework, an integrated system that coordinates screening for psychiatric disorders, neurodevelopmental conditions such as autism spectrum disorder, developmental delay, and psychosocial risk factors, followed by an initial clinical assessment, comprehensive diagnostic evaluation, and longitudinal follow-up. Comprehensive details of the EPSIM procedure are provided in Appendix A.

### 2.2. Participants and Sampling Procedure

A video-based training program was initially developed as part of a nationwide initiative to strengthen healthcare workers’ (HCWs) awareness of ASD. The first implementation of this training was delivered to HCWs in Erzurum, the designated pilot site for EPSIM in Türkiye. Within the EPSIM framework, following the video-based training program, the Child Development Monitoring and Family Support Unit (CDMFSU) unit coordinator contacted 93 of the 126 general practitioners working in 17 of the 23 Family Medicine Units in Erzurum between January 2016 and November 2017. Appointments for children aged 18-48 months under the responsibility of these practitioners were scheduled by the CDMFSU secretary, and assessments were completed for a total of 1981 children, who were subsequently included in the study sample. This section provides an overview of the assessment procedures applied to these 1981 children.

A total of 1981 children were included in the study, comprising 933 girls (47.1%) and 1047 boys (52.9%), with a mean age of 3.09 years (SD = 1.14). The mean age of mothers was 32.06 years (SD = 5.46), and the mean age of fathers was 36.17 years (SD = 5.94). The majority of mothers (74.8%, *n* = 1481) were homemakers. The detailed sociodemographic characteristics of the children are summarized in Table 1.

A total of 1981 children were screened for ASD using the Modified Checklist for Autism with Follow-Up Interview (M-CHAT-R/F). The M-CHAT-R/F questionnaire was completed by the mothers of the participants under the supervision of HCWs. Children who screened positive underwent an initial clinical assessment, which included an unstructured clinical interview with the family and a structured observation of parent-child interactions in a mirrored playroom. Based on the integrated findings from the screening and initial clinical assessment, the consulting child and adolescent psychiatrist identified children at risk for ASD and referred them from the CDMFSU to the NDDMU for comprehensive diagnostic evaluation.

At the Neurodevelopmental Disorders Diagnostic and Monitoring Unit (NDDMU), children identified as at risk for ASD underwent detailed developmental history-taking and DSM-5-TR-based diagnostic evaluations conducted by expert academicians. Children diagnosed with ASD, as well as those classified as at risk, participated in the follow-up program and underwent diagnostic evaluations at 3, 9, and 18 months, and again approximately 10 years later, in accordance with the EPSIM longitudinal monitoring protocol. For clarity, the term “10-year follow-up” used throughout the manuscript refers to the approximate time elapsed since each participant’s initial enrollment.

### 2.3. Assessment Tool and Administration

#### Modified Checklist for Autism with Follow-Up Interview (M-CHAT-R/F)

The Modified Checklist for Autism in Toddlers (M-CHAT) is a widely used, parent-report screening instrument consisting of 23 items designed to identify children at risk for ASD. It was originally developed by Robins et al. [30] in 2001 and subsequently revised in 2014 [31] with the inclusion of a follow-up interview to improve specificity and reduce false positives. Each item on the checklist is answered with a binary response (“yes” or “no”). The M-CHAT is designed to be administered by various HCWs, including child psychiatrists, pediatricians, nurses, psychologists, pedagogues, child development specialists, and special education professionals. Importantly, the screening is based solely on parental report, and no direct observation of the child is required during the administration of the tool. The Turkish version of the M-CHAT underwent a validation and reliability study conducted by Yıkgeç et al. in 2015 [32]. A child who fails two or more of these critical items or three or more items in total is classified as “at risk for autism” and is referred for further diagnostic evaluation.

### 2.4. Statistical Analysis

Statistical analyses were performed using IBM SPSS Statistics v29 (IBM Corp., Armonk, NY, USA). The choice of statistical tests was based on the distribution of the variables. Parametric tests (Student’s *t*-test) were applied to normally distributed continuous variables, whereas non-parametric tests (Mann-Whitney U test) were used for variables that did not meet normality assumptions. Categorical variables were evaluated using Pearson’s chi-square test or Fisher’s exact test, as appropriate. All analyses were conducted using a 95% confidence level.

## 3. Results

Among the total sample, 27 children (1.4%) were identified as requiring further evaluation for ASD. Of these at-risk children, 4 (14.8%) were female and 23 (85.2%) were male, with a mean age of 2.79 years (SD = 1.07). The mean ages of mothers and fathers were 30.41 years (SD = 7.52) and 34.88 years (SD = 4.33), respectively. Mean maternal and paternal education levels were 11.92 years (SD = 4.46) and 12.70 years (SD = 4.19), respectively. The sociodemographic characteristics of children identified as at risk for ASD (*n* = 27) were compared with those of children with no identified risk (*n* = 1954), as summarized in Table 2.

Following DSM-5-TR-based diagnostic evaluations of children identified as at risk for ASD, nine children were diagnosed with ASD, representing 33.3% of those at risk and approximately 0.45% of the total sample. During the eighteen-month longitudinal follow-up, all nine children retained their ASD diagnoses, as confirmed by repeated assessments conducted at 3, 9, and 18 months after the initial evaluation.

**Figure 1:**
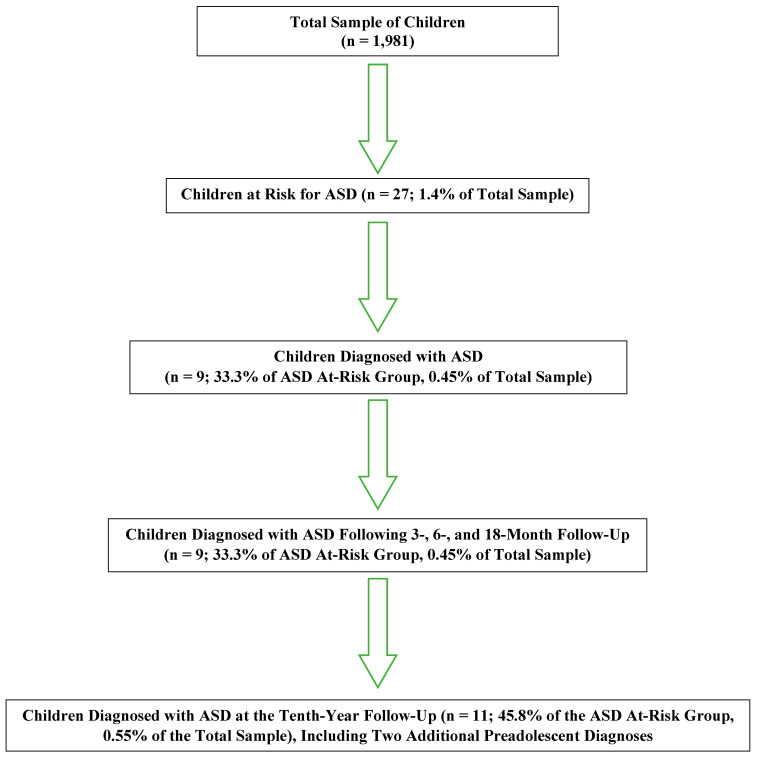
Summary of screening, initial clinical assessments, diagnostic evaluations, and follow-up assessment data for the entire sample.

By the end of the tenth year, 24 children remained in the follow-up program. Two additional children were diagnosed with ASD, bringing the total number of children with ASD to 11, representing approximately 45.8% of those at risk and 0.55% of the total sample. The sociodemographic characteristics of children diagnosed with ASD (*n* = 11) were compared with those of children identified as at risk but not ultimately diagnosed (*n* = 13), as summarized in Table 3. Additionally, the screening, initial clinical assessments, diagnostic evaluations, and follow-up assessment data for the entire sample are summarized in Figure 1.

## 4. Discussion

This prospective, clinic-based cohort study represents one of the most comprehensive longitudinal investigations to date into the diagnostic process, diagnostic stability, and risk trajectory of children identified as at risk for ASD in early childhood in Türkiye. Within the EPSIM framework, a large cohort of 1981 children aged 18-48 months referred from primary care was examined, revealing that 1.4% were identified as at risk for ASD and 0.5% received a confirmed diagnosis based on standardized DSM-5-TR aligned assessments. Notably, all children diagnosed with ASD following the initial assessment retained their diagnosis at the 18-month follow-up, demonstrating strong diagnostic stability throughout early childhood. At the 10-year follow-up, additional diagnoses were observed among children within the original at-risk group during the preadolescent period. Collectively, these findings highlight the critical value of systematic early screening pathways, elucidate meaningful sociodemographic patterns related to referral and diagnosis, and provide key insights for optimizing early identification and service delivery within the Turkish healthcare system.

The proportion of children identified as at risk for ASD in the present study (1.4%) and those who received a confirmed diagnosis (0.5%) are broadly consistent with estimates from community-based toddler screening studies, which report ASD prevalence ranging from approximately 0.3% to 1.0% in similar age groups [33,34]. The rate of ASD diagnosis among children who screened positive (45.8) was found to be higher than the positive predictive values reported in international studies using the M-CHAT-R/F [8,35]. These findings provide robust support for the validity of our approach to identifying at-risk children within the screening framework. Notably, integrating structured observations of parent-child interactions may have enhanced the specificity of the screening process in this study and reduced unnecessary referrals to tertiary services.

One of the notable findings of this study is the complete diagnostic stability observed among the nine children diagnosed with ASD, all of whom retained their diagnosis over the 18-month follow-up period. Diagnostic stability in the toddler and preschool years has been repeatedly demonstrated in high-quality longitudinal studies, particularly when standardized instruments are used [36,37,38]. The stability observed in this cohort suggests reliable early identification and underscores the robustness of the multi-stage evaluation model implemented in the pilot region. These data also support the growing consensus that ASD can be accurately diagnosed as early as 18-24 months when assessments are comprehensive and conducted by trained clinicians [39]. In addition to the high diagnostic stability of ASD observed in early childhood, the subsequent diagnosis of two children approaching early adolescence highlights the clinical variability within the spectrum [22,40]. These findings particularly emphasize the importance of long-term, longitudinal monitoring of children who are at risk but have not yet received a diagnosis.

Sociodemographic comparisons reveal several notable patterns. First, the predominance of male sex among children who screened positive for risk and those diagnosed with ASD aligns with the high male-to-female ratios reported in the international literature [41,42]. This pronounced imbalance suggests that ASD characteristics in girls may manifest differently often in subtler ways resulting in lower detection rates within early screening pathways [43,44,45]. Second, children identified as at risk tended to have fewer siblings and were more likely to be first-born. This pattern may reflect parents’ heightened sensitivity to atypical developmental behaviors in their first-born or greater opportunity for close observation. Comparable associations have been reported in previous epidemiological studies [46,47]. Third, differences in maternal employment status appear relevant for both screening and diagnosis. Compared with mothers of children without developmental risk, mothers of children who were at risk or diagnosed with ASD were less likely to be homemakers. Some evidence indicates that home-centered maternal caregiving may offer contextually protective benefits for child development [48,49], whereas mothers engaged in the workforce may have greater exposure to developmental information, broader professional networks, and a lower threshold for responding to borderline developmental concerns, factors that can facilitate earlier identification and help-seeking for developmental difficulties [50,51]. In this sample, parental health conditions and health-related habits did not differ significantly across groups, suggesting that these factors were unlikely to have influenced the screening or diagnostic outcomes.

## 5. Strengths and Limitations

This study leverages a large, population-based sample representative of a national early childhood cohort and employs a prospective longitudinal design with a 10-year follow-up, enhancing the reliability of developmental trajectory analyses. The use of screening and diagnostic tools within a multi-stage assessment framework integrated into real-world clinical workflows strengthens both the methodological rigor and ecological validity of the study.

Nevertheless, several limitations should be acknowledged. Conducting the study at a single center restricts sample diversity, and the relatively small number of confirmed ASD cases limits the feasibility of multivariable statistical analyses. Additionally, there remains a potential risk of under-identifying females or children with subtler symptom presentations, which may affect the generalizability of the findings.

## 6. Conclusions

The structured, multi-stage model implemented in the pilot region which encompassed healthcare worker awareness training, systematic referrals from FMUs, standardized screening at the CDMFS, and advanced diagnostic assessment at the NDDMU demonstrated both feasibility and effectiveness. This integrated approach is consistent with international recommendations for population-level ASD surveillance systems. This study provides one of the most comprehensive datasets to date on early childhood ASD screening and diagnosis in Türkiye. The findings indicate that a multi-stage early identification model, integrating primary and tertiary care services, is both feasible and effective. The observed high diagnostic stability further underscores the reliability of early ASD diagnosis. Nevertheless, the subsequent identification of additional cases highlights the necessity for long-term monitoring of at-risk children.

Building on this pilot implementation, conducting population-based studies and establishing an effective ASD monitoring system are critical steps for strengthening early intervention strategies and optimizing service planning. The widespread implementation of this structured, multi-stage screening and diagnostic model will enhance the timely identification of children at risk, ensure equitable access to specialist assessments, and support the development of a comprehensive framework for ASD monitoring and care.

## Figures and Tables

**Table 1:** Sociodemographic Characteristics of Children.

Variables	% (*n*)/MEAN ± SD
Child Age (years)	3.1 ± 1.1
Gender	
Male	52.9% (*n* = 1047)
Number of Siblings	2.3 ± 1.1
Birth Order	
First-born	39.8% (*n* = 789)
Maternal Age (years)	32.1 ± 5.5
Paternal Age (years)	36.2 ± 5.9
Maternal Health Status	
Presence of Chronic Illness	13.2% (*n* = 261)
Parental Health Status	
Presence of Chronic Illness	7.3% (*n* = 144)
Maternal Occupation	
Homemaker	74.8% (*n* = 1481)
Household Income Level	
Low	4.0% (*n* = 80)
Maternal Education (years)	10.2 ± 4.5
Parental Education (years)	12.2 ± 3.9
Maternal Health-Related Habit (e.g., smoking, alcohol)	11.8% (*n* = 233)
Paternal Health-Related Habit (e.g., smoking, alcohol)	49.5% (*n* = 981)

SD; Standard deviation.

**Table 2:** Comparison of Sociodemographic Characteristics Between Children at Risk for ASD and Those Not at Risk.

Variables	Children at Risk for ASD (*n* = 27) *n* (%)/MEAN ± SD	Children Not at Risk for ASD (*n* = 1954) *n* (%)/M ± SD	*p* Value
Child Age (years) ^a^	2.8 ± 1.1	3.1 ± 1.1	0.162
Gender ^b^			
Male	23 (85.2%)	1024 (52.4%)	**<0.001**
Number of Siblings ^a^	1.7 ± 0.7	2.3 ± 1.1	**<0.001**
Birth Order ^b^			
First-born	16 (59.3%)	773 (39.9%)	**0.044**
Maternal Age (years) ^a^	30.4 ± 7.5	32.1 ± 5.4	0.114
Paternal Age (years) ^a^	34.8 ± 4.3	36.2 ± 5.9	0.264
Maternal Health Status ^b^			
Presence of Chronic Illness	3 (11.1%)	258 (13.4%)	0.733
Paternal Health Status ^b^			
Presence of Chronic Illness	1 (3.7%)	143 (7.4%)	0.463
Maternal Occupation ^b^			
Homemaker	13 (48.1%)	1468 (76.1%)	**0.002**
Household Income Level ^b^			
Low	2 (7.4%)	78 (4.0%)	0.126
Maternal Education (years) ^a^	11.9 ± 4.5	10.2 ± 4.5	**0.043**
Paternal Education (years) ^a^	12.7 ± 4.2	12.2 ± 3.9	0.520
Maternal Health-Related Habit ^b^ (smoking/alcohol)	5 (18.5%)	228 (11.8%)	0.211
Paternal Health-Related Habit ^b^ (smoking/alcohol)	13 (48.1%)	968 (50.2%)	0.491

SD; Standard deviation, ^a^ Mann-Whitney U test, ^b^ Chi-squared test.

**Table 3:** Comparison of Sociodemographic Characteristics Between ASD-Diagnosed and At-Risk Non-Diagnosed Children.

Variables	Children at risk for ASD (*n* = 11) *n* (%)/MEAN ± SD	Children Not at Risk for ASD (*n* = 13) *n* (%)/M ± SD	*p* Value
Child Age (years) ^a^	2.4 ± 0.6	3.2 ± 1.3	0.434
Gender ^b^			
Male	10 (90.9%)	12 (92.3%)	0.904
Number of Siblings ^a^	1.7 ± 0.6	1.84 ± 0.80	0.817
Birth Order ^b^			
First-born	6 (54.5%)	8 (61.5%)	0.977
Maternal Age (years) ^a^	32.7 ± 6.1	28.8 ± 8.1	0.322
Paternal Age (years) ^a^	35.5 ± 3.9	34.2 ± 4.8	0.617
Maternal Health Status ^b^			
Presence of Chronic Illness	1 (9.1%)	1 (7.7%)	0.904
Paternal Health Status ^b^			
Presence of Chronic Illness	1 (9.1%)	0 (0%)	0.277
Maternal Occupation ^b^			
Homemaker	5 (45.5%)	7 (53.8%)	0.500
Household Income Level ^b^			
Low	2 (18.2%)	0 (0%)	0.551
Maternal Education (years) ^a^	11.9 ± 5.1	12 ± 3.9	0.742
Paternal Education (years) ^a^	12.1 ± 3.8	12.9 ± 4.8	0.439
Maternal Health-Related Habit ^b^ (smoking/alcohol)	2 (18.2%)	3 (23.1%)	0.773
Paternal Health-Related Habit ^b^ (smoking/alcohol)	5 (45.5%)	7 (53.8%)	0.500

SD; Standard deviation, ^a^ Mann-Whitney U test, ^b^ Chi-squared test.

**Figure A1:**
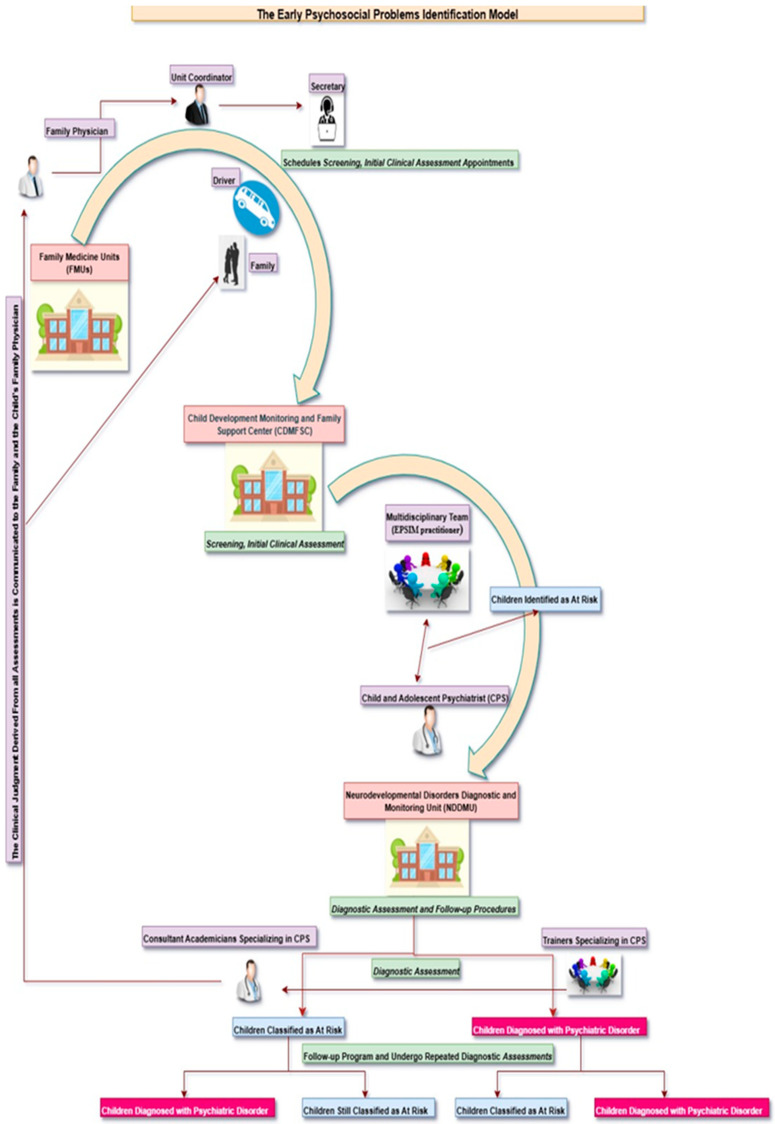
Implementation process of the Early Psychosocial Problems Identification Model (EPSIM).

## Data Availability

The data supporting the findings of this study are not publicly available due to restrictions imposed by the EPSIM protocol and cannot be shared.

## Abbreviations

The following abbreviations are used in this manuscript:

EPSIM	Early Psychosocial Problems Identification Model
ASD	Autism Spectrum Disorder
DSM-5-TR	Diagnostic and Statistical Manual of Mental Disorders, Fifth Edition, Text Revision
M-CHAT-R/F	The Modified Checklist for Autism in Toddlers, Revised with Follow-Up
HCWs	Healthcare Workers
FMUs	Family Medicine Units
CDMFSU	The Child Development Monitoring and Family Support Unit
NDDMU	The Neurodevelopmental Disorders Diagnostic and Monitoring Unit

## Appendix A

### Appendix A.1. EPSIM

The Early Psychosocial Problems Identification Model (EPSIM), established under a protocol between the Atatürk University Faculty of Medicine and the Provincial Directorate of Health, was developed to assess the psychosocial development of children aged 18–48 months, identify risk factors and early indicators of psychiatric disorders, systematically evaluate the parent-child relationship, and facilitate appropriate referral and parent-training procedures. The pilot implementation of EPSIM was conducted in Erzurum, the largest city in northeastern Türkiye and the principal regional healthcare hub, which provides tertiary health services not only to its own population of approximately 750,000 but also to residents of eleven surrounding provinces [52]. This extensive service area, covering a large child population across the province and surrounding regions, provides a high-demand context for evaluating the model’s feasibility and utility.

The EPSIM comprises two distinct units: the Child Development Monitoring and Family Support Unit (CDMFSU) and the Neurodevelopmental Disorders Diagnostic and Monitoring Unit (NDDMU). The CDMFSU includes a waiting area, a multipurpose room for parent-training sessions and team meetings, a mirrored playroom for conducting developmental assessments and observing parent-child interactions, a closed-circuit camera system, and a consultation room for administering forms and assessment scales. The unit is staffed by a multidisciplinary team, including a unit coordinator, an EPSIM practitioner (a general practitioner, physician, nurse, or psychologist trained in EPSIM procedures), a consulting child and adolescent psychiatrist, a secretary, a cleaning staff member, and a driver.

The unit coordinator identifies eligible children through local Family Medicine Units (FMUs) and oversees daily operations, while the unit secretary coordinates screening and initial clinical assessment appointments with families. The driver is responsible for transporting families from the FMUs to the CDMFSU at the scheduled appointment time. The EPSIM-trained practitioner conducts comprehensive 30 min assessments, including developmental testing, screening questionnaires for autism and psychopathology, evaluation of psychosocial, motor, and language development, assessment of behavioral and emotional symptoms, family and medical history, developmental history, parent-child interaction observation, psychosocial risk evaluation, sleep and feeding assessments, toilet training status, hearing and physical development, direct observation. Additionally, the EPSIM practitioner provides guidance on promoting child development, educates parents on the significance of developmental delays, offers recommendations regarding screen time limits, advises on parenting attitudes, and supports the establishment of structured playtime. The consulting child and adolescent psychiatrist conducts a classical unstructured assessment, including a 20 min interview with the family, observation and assessment of the child in the mirrored playroom, and review of screening materials with a member of the screening team, while also developing the annual screening schedule, selecting assessment instruments, supervising EPSIM practitioner training, and reviewing assessment outcomes. All assessment tools are validated, culturally and linguistically adapted, and administered by trained personnel. Assessment results are reviewed by the EPSIM practitioner and the consulting child and adolescent psychiatrist to formulate a clinical judgment, shared with families, and communicated to the child’s family physician. Children requiring further evaluation are referred to the NDDMU for comprehensive diagnostic assessment.

The NDDMU, the tertiary center for comprehensive diagnostic evaluation within EPSIM, is staffed by an academic team consisting of two consultant academicians specializing in Child and Adolescent Psychiatry and eight trainers in the same specialty. The academic team conducts a comprehensive psychiatric assessment based on the Diagnostic and Statistical Manual of Mental Disorders, Fifth Edition, Text Revision (DSM-5-TR) criteria for children referred to the NDDMU, reviews videos of the child during play at home, and evaluates feedback from teachers. Children diagnosed with a psychiatric disorder are referred for appropriate treatment; moreover, all children referred to the NDDMU are monitored within a structured follow-up program and undergo diagnostic evaluations at regular intervals, regardless of whether they receive a psychiatric diagnosis. Figure A1 presents a schematic overview of the procedures implemented within the EPSIM framework.
